# Posterior Estimates of Dynamic Constants in HIV Transmission Modeling

**DOI:** 10.1155/2017/1093045

**Published:** 2017-11-07

**Authors:** Yingqing Chen, Renee Dale, Hongyu He, Quoc-Anh T. Le

**Affiliations:** ^1^Fred Hutchinson Cancer Research Center, Seattle, WA, USA; ^2^Department of Biological Sciences, Louisiana State University, Baton Rouge, LA 70803, USA; ^3^Department of Mathematics, Louisiana State University, Baton Rouge, LA 70803, USA

## Abstract

In this paper, we construct a linear differential system in both continuous time and discrete time to model HIV transmission on the population level. The main question is the determination of parameters based on the posterior information obtained from statistical analysis of the HIV population. We call these parameters dynamic constants in the sense that these constants determine the behavior of the system in various models. There is a long history of using linear or nonlinear dynamic systems to study the HIV population dynamics or other infectious diseases. Nevertheless, the question of determining the dynamic constants in the system has not received much attention. In this paper, we take some initial steps to bridge such a gap. We study the dynamic constants that appear in the linear differential system model in both continuous and discrete time. Our computations are mostly carried out in Matlab.

## 1. Introduction

Patients infected with Human Immunodeficiency Virus (HIV) are very likely to develop Acquired Immunodeficiency Disease Syndrome- (AIDS-) related diseases that are usually fatal if not treated with effective antiretroviral therapies. Since the discovery of HIV in 1983, an efficacious vaccine is yet to be developed to fight the deadly virus. Although highly active antiretroviral therapies (HAART) invented in mid-1990s have saved millions of lives and deterred the disease progression of those infected, HIV infection remains a public health threat. Reducing the risk of HIV transmission is of top priority.

One particular challenge in HIV prevention is its long period of latency period. The average time of an HIV infected patient to become symptomatic with AIDS-related diseases can be more than 10 years [1]. In the sexual transmission of HIV, many of the HIV infected patients may not be aware of their HIV infection status, and the virus continues spreading to their HIV negative partners. Therefore, an in-depth understanding of HIV transmission is the key to successful HIV prevention.

HIV dynamics have long been studied in the field of mathematical epidemiology using linear and nonlinear models [2, 3]. The classic model in epidemiology is the SIR model, which considers the dynamics of the susceptible, infected, and recovered populations [4]. This model is not useful for HIV dynamics, as there is no recovered population. An extension of this is the SEIR model, which includes the population of individuals who are exposed but not yet infected. The period between exposure and infectiousness in HIV lasts about two to four weeks [1]. Since a recovered population does not exist, we can consider this period to have a negligible effect on population dynamics.

Hierarchical models are common in HIV modeling due to the high correlation between risky behavior and HIV incidence [5]. In this paper we will incorporate risk indirectly by considering diagnosed and undiagnosed populations. Intuitively, diagnosed individuals would modify their behavior relative to their behavior prior to the diagnosis.

In this paper, we shall form two models: a continuous time linear differential model and a discrete time differential model. These models are the most fundamental among their kinds. The focus will then be given to determination of the parameter estimates, the dynamic constants in these models. As we will show in this paper, the estimates of the dynamic constants depend on the type of model as well as the qualitative properties of the models.

There are two important dynamic constants in our model, namely, the transmission rates for diagnosed HIV population and for undiagnosed HIV population. One important finding in our study is that the transmission rates for the diagnosed and undiagnosed infected populations are comparable. This leads to our conclusion that the transmission rates should be attached to different groups of susceptibles based on their risk level.

## 2. General Nonlinear Differential Model

One of the frequently used mathematical models for HIV population dynamics can be described as follows. Let *S*(*t*) be the susceptibles. We divide the HIV positive population into two groups: *N*_0_ is the populations that are unaware of the infection; *N*_1_ is the populations that are aware of the infection. Let *ϵ*_*i*_ be the mortality rate for the group *N*_*i*_. Let *r* be the growth rate of the susceptibles. Let *γ*_0_ be the transmission rate of *N*_0_ group and let *γ*_1_ be the transmission rate of *N*_1_ group. Then we have the following nonlinear differential equations:

(1) dStdt=Str−γ0N0t−γ1N1t;dN0tdt=1−βγ1N1tSt+γ0N0St−δN0−ϵ0N0;dN1tdt=βγ1N1tSt+δN0−ϵ1N1.

Here *γ*_1_*N*_1_(*t*)*S*(*t*) counts for those who are infected by group *N*_1_ (per unit time), and among them *β* is the proportion of those who are aware of their infection. The constant *δ* denotes the rate of the HIV positive population in *N*_0_ group who become aware of their infection (per unit time). So there is a flow of *δN*_0_(*t*) from group *N*_0_ to *N*_1_ once a member from *N*_0_ finds out his/her infection through HIV testing.

Many variations of this nonlinear dynamic model have been considered and appeared in the literature to study the HIV population dynamics. For example, in [6], mortality rate of the susceptibles is considered and appears in the differential equation of *S*(*t*). In addition, the parameters are allowed to change but are* piecewise* constant.

In our differential equation model, we have a few constants: *β*, *δ*, *γ*_0_, *γ*_1_, *ϵ*_0_, and *ϵ*_1_. These constants essentially determine the qualitative and quantitative properties of the mathematical model. We shall call these constants the* dynamic constants* of the model. Notice that some of the constants, like *γ*_*i*_, may have prior estimates, based on the data collected directly from the groups *N*_*i*_ and *S*. Some of the constants, like *ϵ*_*i*_, will have posterior estimates. The constants *δ*, *β* may have prior estimates. Our main focus here is to give posterior estimates of these constants.

We shall remark here that the dynamic constants are model-dependent. This might not be obvious. Even though many of them can be estimated statistically without reference to any models, applying these estimates directly to the model may be problematic, as we shall see in the next section. In this paper, we take some initial steps to estimate the model-based dynamic constants.

## 3. Linear Differential Model and Preliminary Discussions

We shall now build a simpler linear model. The main assumption is that the susceptible population is a lot larger than *N*_0_ and *N*_1_. The change of susceptible population, due to HIV infection, is quite small, comparing with the overall size of susceptible. Therefore, we may ignore the dynamics of susceptible population, by assuming that the susceptible population is a constant. This more or less justifies the use of linear system only involving *N*_0_ and *N*_1_.

Let us start with the HIV transmission rate estimates by Pinkerton [7]. The estimates of transmission rates are

(2) γ0≅0.0927,γ1≅0.0268.

*γ*_*i*_ are estimated in terms of infection transmitted per person per year. Since the overall susceptible population is a lot larger that *N*_*i*_, we can assume that HIV transmission events are proportional to the size of *N*_1_ and *N*_0_. Based on this hypothesis, we may model HIV transmission by linear differential equations:

(3) dN0dt=1−βγ1N1+γ0N0−δN0−ϵ0N0,dN1dt=βγ1N1+δN0−ϵ1N1.

The dynamic constants *δ* and *β* remain unchanged. It is also known that *ϵ*_1_≅1.9% [8]. There is no statistics done on *ϵ*_0_. So we can assume *ϵ*_0_≅1.9% as well.

Next, we shall apply the known estimates and study our linear differential model. Notice that *β* remain unknown at this moment. According to [8], *δ* is somewhere around 1/4. We may tentatively set *δ* = 1/4. Utilizing the estimates of dynamic constants directly from [7, 8], let us consider several cases.

### 3.1. *β* = 4/5

We start by assuming that *β* takes the value of the overall portion of those who are aware of their infection. Now we have the following linear equations:

(4) dN0dt=0.02685N1+0.0927N0−14N0−0.019N0.dN1dt=0.0268×45N1+14N0−0.019N1.

We found that the two linear independent solutions have growth rate of

(5) λ+=0.01,λ−=−0.18.

However, we know that the growth of *N*_0_ + *N*_1_ is about 0.048. Hence our assumption *β* = *η* = 4/5 is not valid. Even if we ignore the mortality rate, we have

(6) λ+=0.021,λ−=−0.16.

This is still far below the estimated 4.8% growth rate.

### 3.2. *β* = 1 or *β* = 0

One extreme is that *β* = 1, meaning that the population infected by *N*_1_ gets tested and becomes aware of their infection (within the first year). We have

(7) dN0dt=0.0927N0−14N0−0.019N0,dN1dt=0.0268N1+14N0−0.019N1.

Under this assumption *N*_0_ will decrease at the rate of −0.268, which means that the population *N*_0_ will gradually vanish in a few years. This cannot be true.

Another extreme is that *β* = 0, meaning that the population infected by *N*_1_ will be initially unaware of their infection (within the first year). We have

(8) dN0dt=0.0268N1+0.0927N0−14N0−0.019N0,dN1dt=14N0−0.019N1.

We have

(9) λ+=0.016,λ−=−0.211.

The overall HIV population growth will be less than 0.016. This is quite small comparing with the estimate that the growth rate is about 0.048.

### 3.3. *δ*, *β* Not Fixed

One might conclude that *δ* must be a much smaller number than 1/4, what we have initially assumed. We let *δ* and *β* be unfixed. In this case, we have

(10) dN0dt=0.0927−δN0+1−β0.0268N1−0.019N0,dN1dt=0.0268βN1+δN0−0.019N1.

We have the matrix

(11) $$\begin{array}{c} A=\left(\begin{array}{cc} 0.0927-\delta -0.019 & \left(0.0268\right)\left(1-\beta \right)\\ \delta & 0.0268\beta -0.019 \end{array}\right). \end{array}$$

We know the growth rates are controlled by the eigenvalues of *A*. In particular, we might assume that det⁡(*A* − *λ*) = 0 with *λ* = 0.048. This will guarantee that the dominant term of the solution will grow at the rate of 0.048 (per year). Hence we obtain

(12) $$\begin{array}{c} \left(\;0.0257-\delta \;\right)\left(\;0.0268\beta -0.067\;\right)=0.0268\left(\;1-\beta \;\right)\delta . \end{array}$$

Simplifying it, we have

(13) $$\begin{array}{c} 1.56\delta +0.0268\beta ≅0.067. \end{array}$$

Since 0 ≤ *β* ≤ 1, we find that 0.043 ≥ *δ* ≥ 0.0258. This suggests that there are between 2% to 5% of *N*_0_ getting tested. This percentage seems to be too low comparing with the CDC estimate of about 25%.

We shall remark that our discussion is based on the estimates that *γ*_0_ = 0.0927 and *γ*_1_ = 0.0268 [7]. As we have seen, directly using these estimates as dynamic constants in differential equation modeling will be inadequate to produce the right kind of outcomes and trend. In this paper, we shall discuss posterior estimate of parameters and hope to find some remedy.

## 4. Posterior Estimate of Parameters

In our earlier discussion, we directly insert the transmission rates from the statistical analysis into the linear differential system. The result is not satisfactory. It is desirable to estimate the transmission rates that will produce the right kind of outcome from the linear differential system model. Let us recall the CDC data from 2007 to 2013 (in thousands) [8].

We first simplify our notation. Let $N=\left(\begin{array}{c} N_{1}\\ N_{0} \end{array}\right)$. We rewrite our linear system as

(14) $$\begin{array}{c} \frac{dN}{dt}=MN\left(\;t\;\right), \end{array}$$

where

(15) $$\begin{array}{c} M=\left(\begin{array}{cc} \beta \gamma_{1}-\epsilon_{1} & \delta \\ \left(1-\beta \right)\gamma_{1} & \gamma_{0}-\delta -\epsilon_{0} \end{array}\right). \end{array}$$

The general solution to this system is

(16) $$\begin{array}{c} N\left(\;t\;\right)=P\exp\lambda_{1}t+Q\exp\lambda_{2}t. \end{array}$$

Here *λ*_1_, *λ*_2_ are eigenvalues of *M*. They can be both real or complex. There is also a degenerate case *λ*_1_ = *λ*_2_ that we do not treat here. The behavior of the linear differential system is quite different in these two cases. It is not surprising that we need to use two different methods to estimate the matrix **M**.

### 4.1. *λ*_1_, *λ*_2_ Real: Simple Curve Fitting

We try a global optimization curve fitting using Matlab. We have

(17) Nt=14.1279.86exp−0.1919t+892.3115.57exp⁡0.0273t.

Let $A=\left(\begin{array}{cc} 14.12 & 892.3\\ 79.86 & 115.57 \end{array}\right)$. Then

(18) M≅A−0.1919000.0273A−1=0.032−0.0400.0291−0.197.

Notice that the dominant term $\left(\begin{array}{c} 892.3\\ 115.57 \end{array}\right)\exp0.0273t$ suggested the overall rate of growth of HIV infected population grows at the rate close to 2.73%. This seems to be reasonable. But *δ*, the rate of flow of population from *N*_0_ to *N*_1_, is estimated at −4%. This is completely off the mark. One remedy is that we first estimate the dominant term and then estimate the remainder.

### 4.2. *λ*_1_, *λ*_2_ Real: Dominant Term Estimate

Suppose that *λ*_2_ < *λ*_1_. Then **P**exp⁡*λ*_1_*t* is the dominant term. We shall have

(19) $$\begin{array}{c} \left\|\;N\left(\;t\;\right)\;\right\|≅\left\|\;P\;\right\|\exp\lambda_{1}t. \end{array}$$

Now

(20) Nt=947.3,973.3,997.1,1021.1,1044.3,1069.4,1092.5.

Using curve *F*(*t*) = *ae*^*λt*^ to fit this data, we obtain

(21) a=927.7,λ=0.0236.

### 4.3. *λ*_1_ Dominant, *λ*_2_ Real

Now we can assume *λ*_1_ = 0.0236 and use curve fitting to find *λ*_2_, **P**, and **Q**. We have

(22) P=922.1115.9,Q=−20.477.6,

and *λ*_2_ = −0.172, *λ*_1_ = 0.0236. It follows that

(23) M=βγ1−ϵ1δ1−βγ1γ0−δ−ϵ0=0.01730.04980.0238−0.1656.

We derive that *γ*_1_ − *ϵ*_1_≅0.041 and *δ*≅0.05. These parameters seem to be reasonable. However, *γ*_0_ − *ϵ*_0_ = 0.0498 − 0.1656 = −0.1158. Hence *γ*_0_ will be a negative number which is not possible.

### 4.4. *λ*_1_, *λ*_2_ Complex with Fixed Real Part

Suppose that *λ*_1_ and *λ*_2_ are complex. Then *λ*_1_ and *λ*_2_ are conjugate to each other. In particular, the real part of *λ*, *ℜ*(*λ*_1_) = *ℜ*(*λ*_2_) should be approximately 0.0236. Write *λ*_1_ = *λ*_0_ + *iμ*. We should have

(24) $$\begin{array}{c} N\left(\;t\;\right)=P\exp\lambda_{0}t\cos\mu t+Q\exp\lambda_{0}t\sin\mu t, \end{array}$$

where *μ* is sometimes called a phase constant. A simple curve fitting shows that

(25) P=904.12182.7,Q=−117420

and *λ*_0_ = 0.0236 and *μ* = −0.017. Hence

(26) M≅P,Q0.02360.017−0.0170.0236P,Q−1=0.01870.0325−0.0089−0.0285.

Let us see what this tells us. We have

(27) M=βγ1−ϵ1δ1−βγ1γ0−δ−ϵ0=0.01870.0325−0.0089−0.0285,γ1−ϵ1=0.0187−0.0089≅0.01,γ0−ϵ0=0.004,δ=0.0325.

This roughly says that there are about 3.25% of *N*_0_ that become aware of their infection every year. The annual transmission rate for *N*_1_ is 2.9%. The annual transmission rate for *N*_0_ is 2.3%.

### 4.5. *λ*_1_, *λ*_2_ Complex

We finally use Matlab global optimization to fit the data in the curve

(28) $$\begin{array}{c} N\left(\;t\;\right)=P\exp\lambda_{0}t\cos\mu t+Q\exp\lambda_{0}t\sin\mu t. \end{array}$$

We obtain *λ*_0_ = 0.0088, *μ* = −0.036,

(29) P=902.4184,Q=−476.3149.5.

Hence we obtain the estimate

(30) M≅902.4−476.3184149.50.00880.036−0.0360.0088902.4−476.3184149.5−1=−0.00650.1684−0.00910.0241.

Now we have

(31) βγ1−ϵ1≅−0.0065,δ≅0.1684,1−βγ1≅−0.0091,γ0−δ−ϵ0≅0.0241.

It follows that

(32) γ1≅ϵ1−0.0156≅0.0034,γ0=0.193+ϵ1≅0.21.

So *γ*_1_ is neglectable and *γ*_0_ is about 21%. This again makes the model invalid.

### 4.6. Discussion

In this section, we choose dynamics constants to fit the temporal data. We have found that these dynamic constants depend on the qualitative properties of the model. Yet, none of the dynamic constants we choose match perfectly with the existing estimates. One reason is that yearly data is not suitable for a continuous time model. Therefore, we shall explore the discrete time model.

## 5. Discrete Dynamic Model

We may regard **N**_*t*_ (*t* = 1,2, 3,4, 5,6, 7) as a discrete time dynamical system. Let us assume that this discrete dynamics is defined by a transition matrix **T**:

(33) $$\begin{array}{c} N_{t+1}=TN_{t}. \end{array}$$

In principle, based on our earlier discussion,

(34) $$\begin{array}{c} T=I+\left(\begin{array}{cc} \beta \gamma_{1}-\epsilon_{1} & \delta \\ \left(1-\beta \right)\gamma_{1} & \gamma_{0}-\delta -\epsilon_{0} \end{array}\right). \end{array}$$

Now we would like to estimate **T**.

### 5.1. Basic Estimates

The easiest way to find **T** is by considering the following matrix equations:

(35) $$\begin{array}{c} \left[\;N_{i}N_{i+1}\;\right]=T\left[\;N_{i-1}N_{i}\;\right]. \end{array}$$

For example, for *i* = 2, we will have

(36) $$\begin{array}{c} \left(\begin{array}{cc} 956.9 & 982.4\\ 178.1 & 170.6 \end{array}\right)=T\left(\begin{array}{cc} 929.3 & 956.9\\ 183.7 & 178.1 \end{array}\right). \end{array}$$

Then we find the following estimate of **T**:

(37) $$\begin{array}{c} \left(\begin{array}{cc} 0.9775 & 0.2641\\ -0.0370 & 1.1568 \end{array}\right)\\ \left(\begin{array}{cc} 1.0118 & 0.0800\\ 0.0302 & 0.7959 \end{array}\right)\\ \left(\begin{array}{cc} 0.9922 & 0.1928\\ 0.0370 & 0.7563 \end{array}\right)\\ \left(\begin{array}{cc} 1.0481 & -0.1482\\ 0.0318 & 0.7880 \end{array}\right)\\ \left(\begin{array}{cc} 0.9427 & 0.5214\\ 0.0639 & 0.5844 \end{array}\right). \end{array}$$

We can see some consistency among these transition matrices. For example, the (2,1)-th entry has been around 3%. This translates into

(38) $$\begin{array}{c} \left(\;1-\beta \;\right)\gamma_{1}≅3\%. \end{array}$$

This is the rate of transmission for group *N*_1_. It seems to be consistent with the estimate of [7].

### 5.2. (Arithmetic) Average Estimate of **T**

Now we may average all **T**'s and obtain

(39) $$\begin{array}{c} T≅\left(\begin{array}{cc} 0.9945 & 0.1820\\ 0.0252 & 0.8163 \end{array}\right). \end{array}$$

Hence

(40) M=βγ1−ϵ1δ1−βγ1γ0−δ−ϵ0=−0.00550.18200.0252−0.1837.

Our estimate yields that *δ*≅18%; in other words, about 18% of those unaware of their infection will become aware of their infection next year. We also have

(41) $$\begin{array}{c} \gamma_{0}-\epsilon_{0}=\delta +\left(\;\gamma_{0}-\delta -\epsilon_{0}\;\right)=-0.0017. \end{array}$$

If the mortality rate *ϵ*_0_ is set to be 0.019, then we have *γ*_0_ = 0.017. Similarly, we have

(42) $$\begin{array}{c} \gamma_{1}-\epsilon_{1}=\beta \gamma_{1}-\epsilon_{1}+\left(\;1-\beta \;\right)\gamma_{1}≅0.02. \end{array}$$

If the mortality rate *ϵ*_1_ is set to be 0.019, then we have *γ*_1_ = 0.039. This suggests that the transmission rate of *N*_1_ group is twice as large as the transmission rate of *N*_0_ group. There may be some truth to it. However, we believe that this estimate is off the mark due to the reason that [**N**_*i*_**N**_*i*+1_] are correlated with each other. Hence each estimate **T** will be biased. We shall correct this and give a more robust estimate later.

### 5.3. Least Square Estimate of **T**

Perhaps a good way to estimate **T** is the least square method. We write

(43) $$\begin{array}{c} \left[\;N_{2}N_{3}\cdots N_{7}\;\right]=T\left[\;N_{1}N_{2}\cdots N_{6}\;\right]. \end{array}$$

Applying the least square method, we find that the least square solution to **T** is

(44) $$\begin{array}{c} \left(\begin{array}{cc} 1.0013 & 0.1406\\ 0.0245 & 0.8350 \end{array}\right). \end{array}$$

This estimate seems to be better than the arithmetic average, in the sense that, irregularities will have smaller effect on the least square solution. Because we can reorder **N**_*i*_'s and the least square solution does not change, we also avoid the pitfall that **N**_*i*_ and **N**_*i*+1_ are correlated. We have our posterior estimates:

(45) M≅βγ1−ϵ1δ1−βγ1γ0−δ−ϵ0=0.00130.14060.0245−0.1650.

This estimate is similar to the arithmetic average we just computed. The dynamic constant estimates will be very similar. We shall then look for a solution that is more robust. One particular reason that the least square estimate is not satisfactory is that there are additional relations like

(46) $$\begin{array}{c} N_{i+k}=T^{k}N_{i} \end{array}$$

that least square method does not take into consideration. In other words, **T**^2^, **T**^3^ can also be estimated and shall be taken into consideration when we estimate **T**. We shall offer one remedy that avoids this issue.

### 5.4. A More Robust Estimate

One of the problems with our estimate is that **N**_*t*_ and **N**_*t*+1_ are correlated to each other. As a remedy, we pick **N**_1_ and **N**_6_ as far from each other as possible. We observe that

(47) $$\begin{array}{c} T\left[\;N_{1}N_{6}\;\right]=\left[\;N_{2}N_{7}\;\right]. \end{array}$$

We compute

(48) $$\begin{array}{c} T=\left[\;N_{2}N_{7}\;\right]{\left[\;N_{1}N_{6}\;\right]}^{-1}=\left(\begin{array}{cc} 0.9965 & 0.1681\\ 0.023 & 0.8533 \end{array}\right). \end{array}$$

Then

(49) M=βγ1−ϵ1δ1−βγ1γ0−δ−ϵ0≅−0.00350.16810.023−0.1467.

We have

(50) δ≅0.1681,γ0−ϵ0≅0.0214,γ1−ϵ1≅0.0195.

### 5.5. Discussion

This estimate of **M** is quite consistent with the least square estimate. Our estimate seems to suggest that the transmission rates for *N*_0_ and *N*_1_ may be in the similar range. By [8], assume that *ϵ*_0_ = *ϵ*_1_ = 0.019. We have

(51) γ1=0.0385,γ0=0.04,δ=0.185;β=βγ1γ1=0.019−0.00350.0385=0.4.

**Figure 1:**
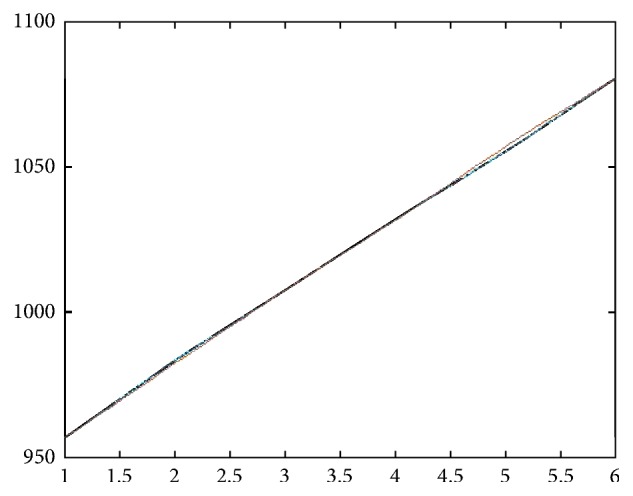
Comparison of **TN**_*t*_ and **N**_*t*+1_: *N*_1_ group.

**Figure 2:**
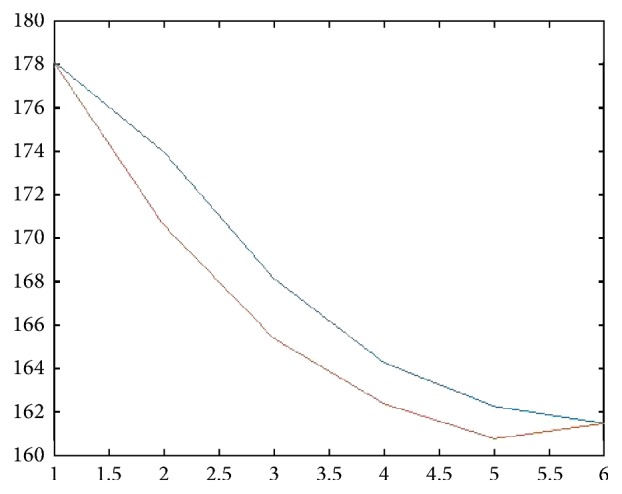
Comparison of **TN**_*t*_ and **N**_*t*+1_: *N*_0_ group.

Every year about 18.5% of those unaware of their HIV positiveness become aware of their infection due to testing. About 40% of those infected by *N*_1_ group become aware of their infection. This seems to be consistent with some of the observations in [7], with one exception; namely, in our estimates, the transmission rates for *N*_1_ and *N*_0_ are very close. Figures 1 and 2 show the difference between **TN**_*t*_ and **N**_*t*+1_.

### 5.6. Arithmetic Average versus Geometric Average

Now we may state our problem in greater generality. Given a temporal vector **N**(*t*), suppose that **N**(*t* + 1) = **TN**(*t*) with transitional matrix **T**. How should one estimate the matrix **T**?

As we discussed earlier, we can use least squares with the equations

(52) $$\begin{array}{c} N\left(\;t+1\;\right)=TN\left(\;t\;\right). \end{array}$$

The least square estimate of **T**, in some sense, is very similar to the arithmetic mean of the transitional matrix **T**. But what makes better sense is a geometric mean. More precisely, we have to take into consideration that

(53) $$\begin{array}{c} N\left(\;t+k\;\right)=T^{k}N\left(\;t\;\right). \end{array}$$

Suppose that **T**_*t*_ is the transitional matrix at time *t*. Then a good estimate of **T** should be the “geometric average” of **T**_*t*_. For scalars, one can define the geometric average of *p*_1_, *p*_2_,…, *p*_*n*_ to be the *n*th root of ∏*p*_*i*_. But matrix multiplications are not commutative and one cannot define the geometric average of matrices. It remains a challenging problem to define computationally a geometric mean of **T**_*t*_.

### 5.7. Roots Estimate

Tentatively, we may define the geometric mean by taking roots. For example, we may now consider

(54) $$\begin{array}{c} T^{2}\left[\;N_{1}N_{4}\;\right]=\left[\;N_{3}N_{6}\;\right]. \end{array}$$

Then

(55) T≅1.00380.12770.02280.8443,M≅0.00380.12770.0228−0.1557.

We can also consider

(56) $$\begin{array}{c} T^{4}\left[\;N_{1}N_{3}\;\right]=\left[\;N_{5}N_{7}\;\right]. \end{array}$$

We have

(57) T=1.00080.14300.03010.8011,M≅0.00080.1430.0301−0.2.

Both estimates of **T** are consistent with the least square estimate and the estimates in the previous section. Above all, all our estimates point to the same range of transmission rates for both *N*_0_ and *N*_1_.

## 6. Concluding Remarks

Now we shall compare our dynamic constant estimates in the linear differential model in continuous time and in discrete time.

In the continuous time model, we obtain the transmission rate *ϵ*_1_ of about 4% for the *N*_1_ group, those who were aware of their HIV infection. Nevertheless, *δ* the rate of flow from *N*_0_ to *N*_1_ due to HIV testing turned out to be too low and *ϵ*_0_ often came out to be negative, which cannot be the case. The best results are obtained when we assume the two eigenvalues are complex. In this case

(58) γ1≅0.029,δ≅0.0325,γ0≅0.023.

Yet *δ* seems to be quite low. According to the CDC report [8], *δ* is estimated at about 25%.

There are various reasons why our dynamic constants are inconsistent with known estimates. Firstly, the CDC data we use tends to underestimate the *N*_0_ and *N*_1_ population sizes, particularly in more recent years. The CDC estimates the sizes of the populations infected with HIV by back calculation. The estimates for any given year will increase as new diagnoses are obtained. HIV may go undiagnosed for up to 10 years without causing the death of the patient (Table 1) [5]. Depending on the stage of the disease, the individual will be counted as undiagnosed for a number of years prior to the diagnosis. This causes the estimates of the population sizes to be smaller than the actual size of the population. A new estimate of the HIV prevalence agrees with this conclusion [9]. Although this new estimate is more conservative than the back calculation method, it may still underestimate the *N*_0_ population. Both estimates show a downward trend in the data, but this is likely to be erased as more individuals are diagnosed in the later stages of the disease.

Secondly, our computations assume that the susceptible population is much larger than the infected population. However, failing to obtain the right estimates suggests that opposite may be true—the susceptible population could be much smaller. HIV infection is overrepresented in some subpopulations, such as men who have sex with men (MSM) [1]. This subpopulation is only about 5% of the US population, or approximately 15 million individuals. Not all MSM can be considered to have equal risk of contracting the disease, and since over 1 million individuals are currently living with the disease, the susceptible population may be comparable to the size of the infected population. For this reason, any differential system model of the HIV transmission must include the susceptible population.

The discrete dynamics model seems to be most robust against the bias caused by back calculation and the size of the susceptibles. By ignoring the 2013 year's data, we are able to determine the transmission rates as

(59) γ1≅0.0385,γ0≅0.04.

Also *δ*≅0.184, not too different from the CDC estimate 0.25. We see that the dynamic constants in the discrete time model are less affected by the underestimation caused by back calculation. It is also true that the relative size of susceptibles has less effect on the discrete time model than on the continuous time model.

Finally, our estimates of the transmission rates for diagnosed and undiagnosed HIV population are relatively close. This is very different from the previous estimate, where the transmission rate of the undiagnosed population is about 4 times as high as the diagnosed population [7]. This implies that the transmission rates should be attached to the susceptible population. It makes sense to divide the susceptible population into groups depending on the possible transmission rates for them. We shall pursue this in a different paper.

## Figures and Tables

**Table 1:** Population of undiagnosed individuals with HIV from 2007 to 2013.

Year	Diagnosed	Undiagnosed	Percentage of total
2007	929.3	183.777	16.5
2008	956.9	178.1165	15.7
2009	982.4	170.6282	14.8
2010	1007.6	165.3507	14.1
2011	1031.6	162.4248	13.6
2012	1057.2	160.7760	13.2
2013	1080.5	161.46	13.0

Source: [8].
